# Application of Biological Glue–Clay Composite Substrate in Slope Ecological Restoration

**DOI:** 10.3390/polym15183763

**Published:** 2023-09-14

**Authors:** Xufen Zhu, Jiaqiang Zheng, Yuliang Gao, Jian Xue, Guochang Hu, Wenyue Che, Zezhuo Song, Jin Liu, Tingwei Huang, Peng Wu

**Affiliations:** 1 School of Earth Sciences and Engineering, Hohai University, Nanjing 210098, China; 2 Jiangsu Institute of Geology and Minerals Investigation, Jiangsu Geological Bureau, Nanjing 210000, China; 3 Jiangsu Shanshui Ecological Environment Construction Engineering Co., Ltd., Nanjing 210018, China; 4 School of Engineering, Royal Melbourne Institute of Technology, Melbourne, VIC 3001, Australia

**Keywords:** bioglue, improved clay, evaporative cracking, scour resistance, micromechanisms

## Abstract

Given the issues of soil cracking, poor water retention during drought, and erosion damage caused by rainfall, we conducted an in-depth study on the water retention properties, cracking resistance, and scouring resistance of biogel-amended clay using evaporation cracking and scouring tests. The hydrophysical properties and cohesive aggregation mechanism of biogel-amended clay were explored, and the results showed that the incorporation of biogel improved the water retention, cracking resistance, and scour resistance of the clay samples. With an increase in the biogel content, the biogel mucous membrane inside the samples improved the cohesion between soil particles, reduced the generation and development of cracks, and improved the cracking resistance. There was no significant cracking of the samples after the biogel content reached 0.3%, which changed the migration of water in the sample, prevented water evaporation, and improved the water retention of the clay samples. Biofilm can change the migration of water in the sample, prevent some evaporation, and reduce the evaporation rate. To a certain extent, it can enhance the water retention capacity of the sample. Enhanced biofilm content significantly reduced scouring in the process of rainfall and runoff erosion of the sample, and biofilm content of 0.2% significantly reduced the surface of the specimen damaged by erosion. The hydrophysical properties of the composite-adhesive-amended clay samples were significantly improved compared with those of the single-bioadhesive-amended clay samples.

## 1. Introduction

Clay is widely distributed in China and naturally occurs in a compact state with high density. The inherent cohesive properties of clay particles and their interactions with water contribute to the high strength and structural stability of the soil [1]. Clay has been used extensively as a fundamental construction material in various engineering applications such as buildings, roads, bridges, and slope protection [2,3].

Owing to the high sensitivity of clay to water, variations in soil moisture content can cause significant changes in its engineering properties. Under extreme weather conditions, clay-related engineering projects are prone to instability owing to fluctuations in the soil moisture content. For instance, during periods of extreme drought, cohesive soil may undergo shrinkage and cracking, which significantly compromise its engineering characteristics and lead to various geological and geotechnical problems. Conversely, under heavy rainfall conditions, soil subjected to rainwater can experience erosion and damage caused by splashing and runoff, thereby affecting the stability of the slopes. In severe cases, this can result in catastrophic natural disasters such as landslides and mudflows, posing risks to human life and properties [4,5]. To mitigate the hazards associated with moisture-induced changes in clay, stabilizers are commonly used to modify its hydraulic properties. Traditionally, methods such as the addition of cement and limestone, among other conventional reinforcement materials, have been used to improve clay. However, these approaches are not environmentally friendly [6,7,8]. In recent years, the concept of sustainable development has gained widespread recognition, prompting researchers to explore the use of environment-friendly materials for clay improvement. Polymer materials have emerged as a relatively novel option for soil modification, offering ecological friendliness, simplicity of application, and stable performance while enhancing clay properties. Researchers are increasingly investigating the applications of polymer materials in this context. Naeini et al. studied the enhancement of the undrained strength in cohesive soil using acrylic-based waterborne polymers [9]. Mousavi et al. conducted bearing ratio tests on clay modified with different proportions of recycled polypropylene (RPP) and found that the modified soil exhibited a significantly reduced expansion pressure and improved strength [10]. Liu et al. employed (STW)- and polyurethane-based polymers to modify the surface soil of different soil types on slopes, resulting in significant improvements in the strength characteristics, water stability, and erosion resistance of cohesive soils [11,12]. Onyejekwe et al. investigated the mechanical properties of clay stabilized with soil stabilizers and observed significant improvements in undrained strength and expansiveness [13]. Zansokhova et al. examined the use of environmentally friendly ionic polymer cement (IPC) binders to help mitigate water and wind erosion in soil and discussed their efficiency and potential scope of application [14]. Liu et al. demonstrated that the swell-consolidation and shrinkage ratios of polyacrylate vinyl acetate copolymer (PAVc)-treated expansive soil were reduced [15]. Mapossa et al. explored the thermal, morphological, and mechanical properties of multifunctional application bentonite composites based on biodegradable polymers [16].

Biogels are natural high-molecular-weight polymers extracted from organic materials such as polysaccharides, humic acids, cellulose, lignin, and other natural substances. Compared to other polymeric materials, biopolymers and their derivatives have unique properties such as being biodegradable, biocompatible, renewable, and having high tensile strength [17]. Over time, they tend to degrade in the environment, leaving a minimal residue. Furthermore, they readily integrate with plant fibers, making them particularly advantageous for enhancing soil properties through biologically induced composite modifications. The application of natural high-molecular-weight polymers in the soil has a smaller environmental impact and is more effective than inorganic additives [18,19]. In recent years, soil improvement has become a popular research topic. Chang et al. [20] treated residual soil with β-1,3/1,6-glucan and observed a significant increase in its undrained compressive and tensile strengths, demonstrating its remarkable economic competitiveness. Cabalar et al. [21] conducted shear characteristic tests on xanthan-gum-modified sandy soil, and the results indicated a positive correlation between the shear strength of the soil and the xanthan gum content. Latifi et al. [22] performed undrained compressive strength tests on peat soil stabilized with xanthan gum. After 28 d of curing, the undrained compressive strength of the stabilized soil with a xanthan gum content of 2% was six times higher than that of untreated soil samples. Sulaiman et al. [23] investigated the modification of soils using different concentrations of xanthan gum and guar gum and found that the addition of a small amount of biogel increased the soil pH, reduced its specific gravity and maximum dry density, and improved the optimum moisture content and plasticity index.

Based on the advantages of biogels in soil improvement and considering the limited research on the hydraulic properties of clay modified by various biogels, this study investigated the synergistic effect of xanthan gum and guar gum in clay modification. Crack and erosion resistance tests were conducted to compare and analyze the hydraulic characteristics of the clay before and after modification. The findings provide a novel approach for improving clay in engineering applications.

## 2. Materials and Methods

### 2.1. Materials

#### 2.1.1. Clay

The clay selected for the test was fine-grained soil obtained from Jiangning District, Nanjing City. Through compaction test, the optimal moisture content *ω*_op_ was 18.94%, and the corresponding maximum dry density *d*_max_ was 1.70 g/cm^3^, liquid–plastic limit of 38.41% and 21.12%, respectively, plasticity index IP of 17.29, and specific gravity of 2.70. According to the USCS classification (ASTM, 2011) [24], soil is classified as lean clay (CL). The chemical compositions of the vegetal soils were analyzed using X-ray diffraction mineralogical tests, and the results are shown in Table 1. The minerals of the tested soil are quartz, kaolinite, and CaO, and the quartz makes up more than half of the mineral composition.

#### 2.1.2. Xanthan Gum

**Figure 1:**
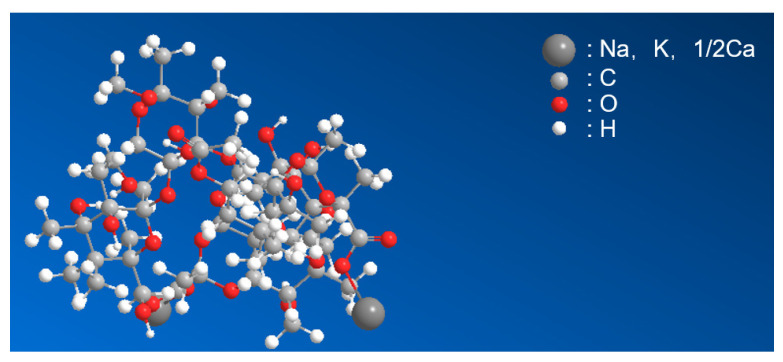
The ball-and-stick model of xanthan gum.

Xanthan gum is a light-yellow to white powder that is easily soluble in water, insoluble in organic solutions, neutral, dispersed in water, and emulsified to form a stable hydrophilic viscous colloid (Table 2). Xanthan gum, purchased from Polifar Group Limited (Nanjing, China), is produced by the fermentation of Xanthomonas campestris, a natural biological polysaccharide composed of D-glucose, D-mannose, and D-glucuronic acid at a ratio of 2:2:1, with a molecular formula of C_8_H_14_C_l2_N_2_O_2_ and a relative molecular mass of 2 × 10^6^–2 × 10^7^. The structural formula is shown in Figure 1. Owing to the long-chain structure of xanthan gum and its numerous functional groups, it has the general properties of long-chain polymers and the following unique properties under specific conditions: (1) suspension and emulsification, (2) water solubility, (3) thickening, (4) pseudoplasticity, (5) thermal stability, (6) acid and alkali resistance stability, (7) salt stability, and (8) stability in enzymatic reactions.

#### 2.1.3. Guar Gum

**Figure 2:**
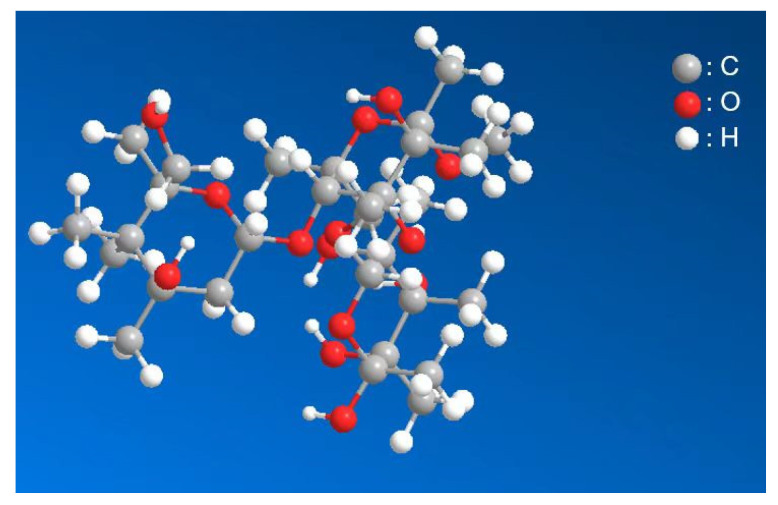
The ball-and-stick model of guar gum.

Guar gum is a white to light-yellow powder that is soluble in water and insoluble in oil, hydrocarbons, ketones, and other organic solvents (Table 3). Guar gum was purchased from Nanjing Yida Biotechnology Co., Ltd. (Nanjing, China), and is a highly purified natural polysaccharide extracted from guar beans, composed of mannose and galactose at a 2:1 ratio, with a relative molecular mass of 2.5 × 10^5^–6.5 × 10^5^; a ball-and-stick model is shown in Figure 2. The unique molecular structural features and naturalness of guar gum give it the following unique properties: (1) water solubility, (2) thickening, (3) pseudoplasticity, (4) salt resistance, (5) acid and alkali stability, and (6) cellulose affinity.

#### 2.1.4. Composite Biogel

Xanthan gum and guar gum molecules contain a large number of hydrophilic groups with strong hydrogen bonds between the molecules, which can significantly improve the viscosity and stability of aqueous solutions or dispersion systems. When xanthan gum and guar gum are mixed, the solution formed has a synergistic effect. Casas et al. [25] analyzed and compared the effects of different xanthan gum and guar gum content ratios and dissolution temperatures on the viscosity of the mixture. The results showed that when the ratio of xanthan gum and guar gum was 3:3, the highest viscosity of mixed solutions of xanthan gum and guar gum was observed at dissolution temperatures of 40 °C and 80 °C. The composite biogel viscosity can reach 1000 MPa·s.

### 2.2. Methods

To study the effect of biogum content on the evaporation cracking process of improved clay, three types of biogums, xanthan gum, guar gum, and a composite of xanthan gum and guar gum with contents of 0.1%, 0.2%, 0.3%, and 0.4%, were used to conduct the evaporation cracking and scouring resistance tests. The biogums and 500 g sieved soil samples were mixed into 50% water content test soil samples according to the design parameters, placed in a square container with a side length of 20 cm and a height of 4 cm, and vibrated uniformly for 5 min to discharge the gases mixed in the specimen during the preparation. The specimen container box was sealed with a lid for 24 h to ensure uniformity of specimen water content and allow the biogas to react. After 24 h, the sample container lid was removed to allow the sample to dry, and the bioglue-amended clay specimens were kept in a well-ventilated air-conditioned room at a constant temperature of 26 °C. After the test, the samples were tested by scanning electron microscopy and X-ray diffraction. The microscopic SEM used in this paper is the SU3500 SEM produced by Hitachi, Tokyo, Japan, with magnification up to 7 nm. The X-ray test instrument is the DX-2700 X-ray diffractometer, produced by Haoyuan Instrument Co., Ltd., Dandong, China; the tube voltage of the instrument is 30 kV, the tube current is 20 mA, and the system parameter—zero point parameter setting—TD: 2.8, TS: 3.52.

#### 2.2.1. Testing of Evaporative Cracking Characterization of Modified Clays

**Figure 3:**
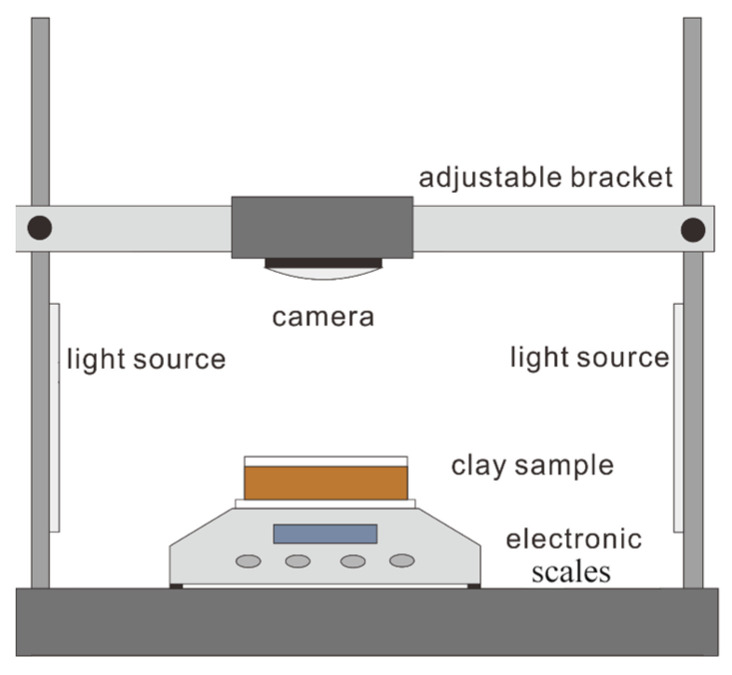
Schematic diagram of the test photography device.

The bottom of the specimen container was covered with 800-mesh sandpaper to simulate the roughness of the interface in the in situ soil. During drying and dewatering, the specimens were weighed at regular intervals using an electronic scale with an accuracy of 0.1 g. The evaporation rate *R*_e_ of the specimens with different biogel contents was calculated based on the water content of the specimens as follows:

(1) $$R_{e}=\frac{m_{t}-m_{t-1}}{t}$$

where *m_t_* is the mass of the specimen weighed at the time *t*; *m_t_*_−1_ is the mass of the specimen weighed before the time *t*; *t* is the time between the two weighing intervals in min. The evaporation curves of the different biogel-amended clay specimens were plotted with respect to time, and the evaporation characteristics of the different biogel-amended clay samples were studied. The development of fissures on the surface of each specimen over time was recorded by photographing the surface of the specimen at a fixed position using a homemade test-filming device, as shown in Figure 3.

#### 2.2.2. Testing of Improved Clay Scour Resistance Properties

**Figure 4:**
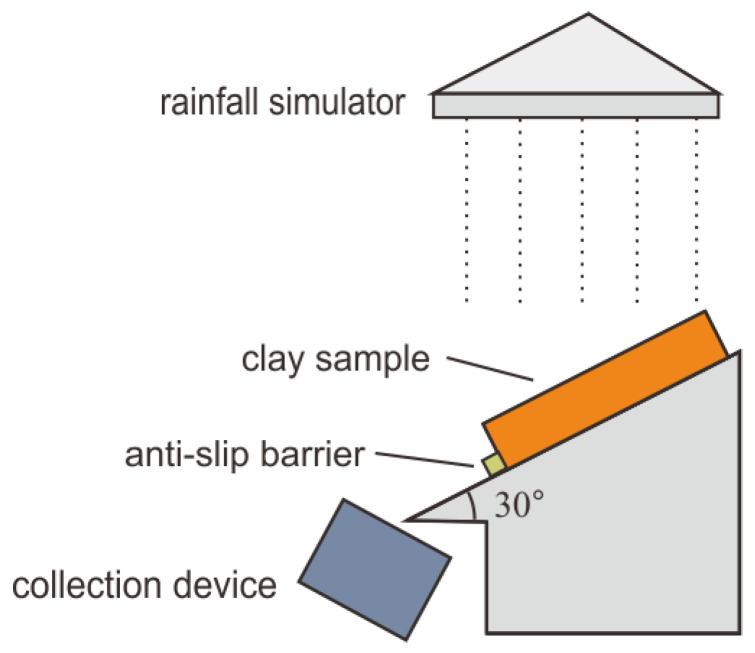
Schematic diagram of the flushing test setup.

During the test, the simulated rainfall intensity was 78 mm/d, reaching the level of heavy rainfall, and the scouring time was 40 min with a slope of 30°. In total, 40 samples were collected, and the mass of dry soil collected per minute was obtained by air-drying and weighing. The mass of dry soil was accumulated over time, and the cumulative dry soil mass scouring characteristic curves were plotted for each time period, which were combined with the scouring damage pattern to analyze and evaluate the scouring characteristics of the biogel-amended clay specimens. When the moisture content of the specimen reached stability, the designed scour simulation device was used to conduct the scour resistance test; a schematic diagram of the test device is shown in Figure 4.

## 3. Results

Soil often undergoes drying, shrinkage, and cracking after evaporation, which reduces its strength and affects engineering applications. Many researchers have studied the factors that affect soil evaporation and cracking. However, there are few reports on the effects of biopolymer modifications on soil evaporation and cracking.

### 3.1. Analysis of Evaporation–Cracking Tests

#### 3.1.1. Analysis of Evaporation Characteristics

**Figure 5:**
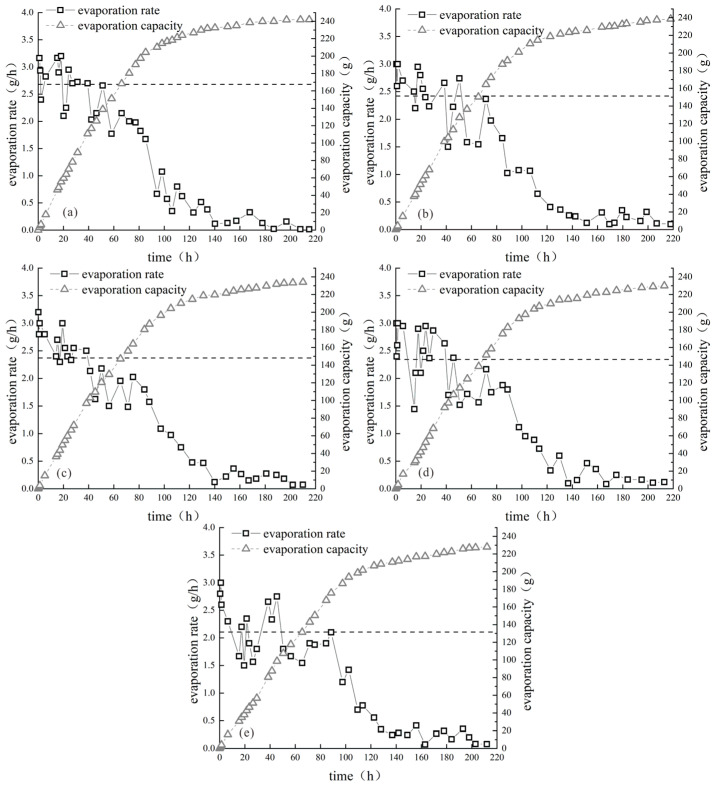
Evaporation curves of improved xanthan gum samples: (**a**) 0%; (**b**) 0.1%; (**c**) 0.2% (**d**) 0.3; (**e**) 0.4%.

**Figure 6:**
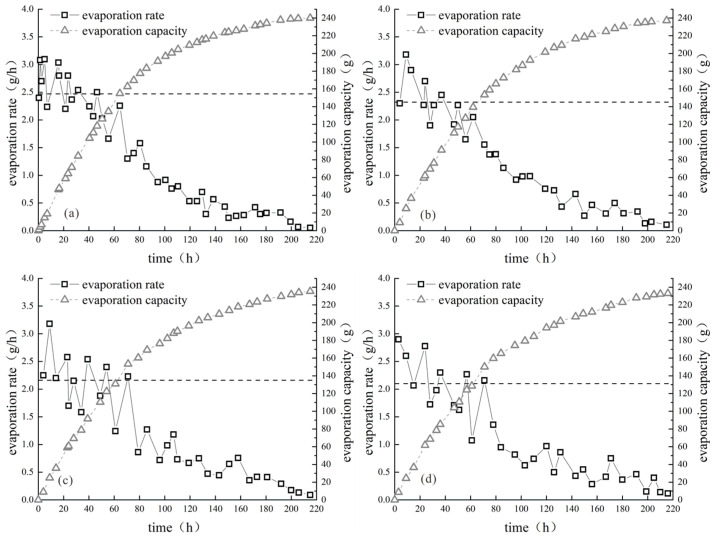
Evaporation curves of modified guar gum samples: (**a**) 0.1%; (**b**) 0.2; (**c**) 0.3%; (**d**) 0.4%.

**Figure 7:**
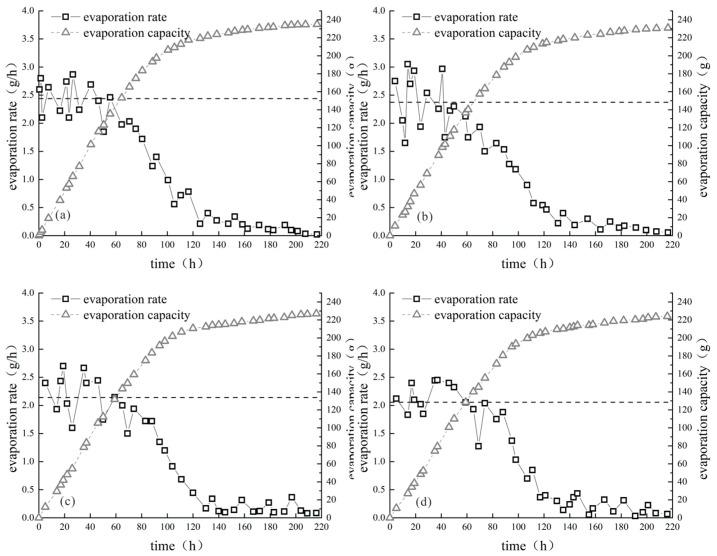
Evaporation curves of composite-gum-modified samples: (**a**) 0.1%; (**b**) 0.2; (**c**) 0.3%; (**d**) 0.4%.

Figure 5, Figure 6 and Figure 7 illustrate the improved evaporation characteristics of clay samples with various biopolymers. Based on the observed trend of the evaporation rate of the sample, the evaporation process can be categorized into three distinct stages: constant speed, deceleration, and stability. To observe the change in evaporation rate with different biopolymer contents, it was essential to calculate the average evaporation rate at the constant-speed stage for each sample and illustrate it as an auxiliary line on the graphs. This approach allows a clear visualization of the relationship between the biopolymer content and evaporation rate.

Figure 5, Figure 6 and Figure 7 show that the evaporation rate curves and trends of the clay samples modified with various biopolymers were similar. The average evaporation rate during the constant-speed stage decreased as the biopolymer content increased, and the amount of evaporation decreased slightly with increasing biopolymer content. For example, the average evaporation rates during the constant-speed stage of modified clay samples with xanthan gum content of 0%, 0.1%, 0.2%, 0.3%, and 0.4% were 2.68 g/h, 2.42 g/h, 2.37 g/h, 2.34 g/h, and 2.11 g/h, respectively, indicating a continuous decrease in the average evaporation rate with increasing xanthan gum content. When the xanthan gum content was 0.4%, the average evaporation rate during the constant-speed stage of the modified clay sample was 78.7% of that of the unmodified sample, indicating a significant reduction in the average evaporation rate owing to the addition of xanthan gum. The reason for the significant reduction in the average evaporation rate with the addition of xanthan gum is that it alters the internal particle pores of the sample, which changes the migration path of water during evaporation. Consequently, the water in the sample required a longer migration time to escape from the surface, thereby maintaining the moisture content of the sample.

Figure 6 clearly shows that the addition of guar gum to the sample significantly reduced the evaporation rate. As the guar gum content increased, the average evaporation rate gradually decreased, and the deceleration stage occurred earlier than that in the xanthan gum samples. This indicates that guar gum and xanthan gum have similar mechanisms of action, where the time required for water to evaporate from the sample increases owing to internal water loss. Mucilage formed by guar gum has a more complex structure, which enhances the bonding degree of soil particles, reduces the development of cracks, and prolongs the migration time of water in the sample.

Figure 7 shows that when the content of the composite biopolymer was low (0.1% and 0.2%), the average evaporation rate of the sample was similar to that of a single biopolymer and not less than that of a single biopolymer. However, when the composite biopolymer content was high (0.3% and 0.4%), the average evaporation rate of the sample was significantly lower than that of a single biopolymer. This may be because the composite biopolymers can interact with each other, and the bonding force between the soil particles is weak when the content is low, leaving more pores in the sample unfilled. As the content increased, the adhesive force of the composite biopolymer mucilage on the soil particles was enhanced, thereby improving the water retention capacity of the sample. This indicates that the addition of biopolymers can significantly reduce the average evaporation rate of the sample, and that different biopolymers have a similar effect on the average evaporation rate of the sample, suggesting that their mechanisms of action are similar.

#### 3.1.2. Analysis of Cracking Characteristics

**Figure 8:**
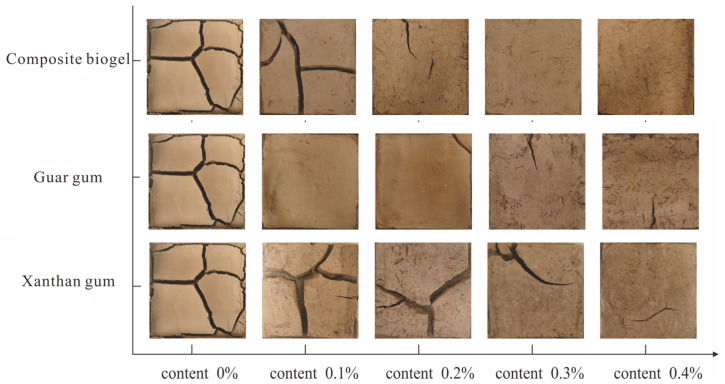
Crack characteristics of biological-glue-modified clay.

Figure 8 shows the crack characteristics of the biological-glue-modified clay samples. The sample was divided into different blocks, and each block exhibited significant shrinkage. When the xanthan gum content was 0.1%, cracks in the sample developed from the edges of the sample towards the center, accompanied by secondary crack development. As the cracks continued to extend, the crack ends reached the edges of the sample and divided the sample into different blocks. When the cracks reached a stable stage, the sample shrank into four blocks with good overall integrity. This is because the addition of xanthan gum enhanced the adhesive effect between the soil particles, resulting in shrinkage and cracking in weakly bonded areas on the surface of the sample after internal water evaporation.

As the xanthan gum content increased, crack development slowed, and the crack width and length gradually decreased with no clear blocks. When the xanthan gum content increased to 0.4%, there was no clear crack development when it reached a stable stage. The crack width decreased, and the sample shrank significantly with good integrity. This indicates that as the xanthan gum content increased, the xanthan gum membrane fully adhered to the soil particles, significantly strengthening the cohesion force between the soil particles in the sample. The tensile stress inside the sample was lower than the cohesive force between the soil particles provided by the xanthan gum membrane, which prevented significant damage to the sample. After moisture evaporated from the sample, pores appeared between the soil particles. Some pores decreased owing to the tensile stress provided by the xanthan gum membrane, whereas others were filled by it. The tensile stress between the soil particles was borne by the xanthan gum membrane, which hindered crack development and maintained the overall integrity of the sample.

The cracking changes of the clay samples improved by guar gum were similar to those improved by xanthan gum, but the overall integrity of the guar-gum-improved samples was higher than that of the xanthan-gum-improved clay samples. The degree of crack development in the guar-gum-improved samples was significantly lower than that in the xanthan-gum-improved clay samples with the same content. It is speculated that the addition of guar gum enhanced the bonding effect between the soil particles in the sample, effectively preventing the occurrence and development of cracks in the sample.

The anticracking properties of the composite-gum-improved clay samples were weaker than those of the guar-gum-improved clay samples at a content of 0.1%. However, when the content was greater than 0.2%, the integrity and anticracking effect of the composite-gum-improved clay samples were greater than those of the single biopolymers. This indicated that composite gum formed composite gum mucilage between the soil particles in the sample. When the content was greater than 0.2%, the degree of soil particle encapsulation by the composite gum mucilage significantly increased, resulting in a significant increase in the cohesive force between the soil particles. Composite gum mucilage forms a three-dimensional network structure that fills the sample and bonds the soil particles, thereby enhancing the overall integrity of the sample. Compared to a single biopolymer, mucilage has a stronger bonding effect, effectively resisting the tensile stress generated during water evaporation and preventing the occurrence and development of cracks, which can maintain the integrity of the sample.

### 3.2. Analysis of Scouring Resistance of Biogel-Improved Clays

#### 3.2.1. Effect of Xanthan Gum Content on the Scouring Amount of Improved Clay 

**Figure 9:**
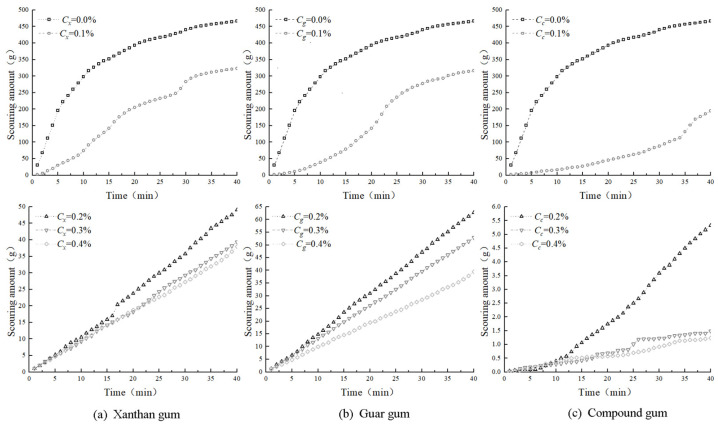
The scouring characteristic curve of biogum-improved clay with different gum contents.

The scouring characteristic curve of the biogum-improved clay with different content percentage is illustrated in Figure 9. For the plain soil sample, the curve is upward convex, where the soil sample is subjected to scouring when scour damage occurs in a short time, which produces a large number of scour products. With time, the residual unwashed portion decreased dramatically, which led to a reduction in the subsequent scour amount of the sample. As shown in Figure 9a, for the clay samples modified with 0.1% xanthan gum content, the curve is almost S-shaped, which indicates that the incorporation of xanthan gum improved the scouring performance, and the characteristics of scour damage and the prescouring of plain soil samples are similar; however, the scouring amount of xanthan-gum-modified clay specimen is clearly smaller than that of the modified clay specimen. However, the scouring amount of the xanthan-gum-modified clay specimens was smaller than that of the plain soil samples. As the scouring continued, the amount of scour tended to stabilize, and when the xanthan gum content reached 0.2%, the amount of scour increased linearly with time, but the amount of scour decreased significantly compared to that of low-content xanthan gum and even plain soil. For the modified clay sample with 0.1% guar gum (Figure 9b), the graphs are similar to those for 0.1% xanthan gum, and the scouring resistance was greatly improved compared to that of the plain soil samples. After the guar gum content reached 0.2%, the amount of scouring of the samples increased linearly with time and decreased with increasing guar gum content. When the composite gum content was 0.1%, the curve of the modified clay specimen was downward convex (Figure 9c), and the scouring resistance was significantly improved compared to both the vegetal soil and the single biogum specimen. After the composite gum content reached 0.2%, the scour volume of the samples increased linearly over time and the scour volume of the samples decreased with an increase in the composite gum content.

Figure 9 shows that the amount of specimen scouring was reduced with the increase in xanthan gum content. Owing to the increase in xanthan gum content, the quality and volume of the xanthan gum mucous membrane in the specimen increased, which can better bond the soil particles in the specimen, rendering the specimen more stable to the impact of rainwater to improve specimen scouring performance. The incorporation of guar gum rendered the specimen better resistant to scouring in the prescouring period, but the degree of scour resistance was slightly lower than that of the xanthan-gum-improved clay specimen; however, the scouring amount of the guar-gum-improved clay specimen was greater than that of the xanthan-gum-improved clay specimen, and the scouring amount of the guar-gum-improved clay specimen eventually tended to stabilize. The final scour amount of the guar-gum-amended clay specimen was also stabilized, and the cumulative scour amount was similar to that of the xanthan-gum-amended clay specimen.

Comparing the scour curves of the three tested groups, the incorporation of the composite adhesive significantly improved the prescouring scouring performance of the specimens, and the degree of scouring performance was significantly better than that of a single-bioadhesive-modified clay specimen. The composite adhesive was mixed into the specimen to generate a composite adhesive film, which can more effectively resist the impact energy of rainwater in the early stage of scouring. When the specimen started to scour rapidly, the degree of scouring was also lower than that of the single-bioadhesive-amended clay specimen. The cumulative scouring of the final composite-adhesive-amended clay specimen was significantly lower than that of the single-bioadhesive-amended clay specimen. After the composite glue content reached 0.2%, the flushing amount was greatly reduced compared to that of the single-biogel-amended clay specimen, which improved the recording accuracy of the flushing amount of the specimen. The difference in the flushing amount at different times increased, resulting in a nearly linear increase in the flushing amount of the specimen with time. The scouring amount of the specimen decreased with an increase in the composite glue content, and the scouring amount of the composite-glue-amended clay specimen was significantly smaller than that of the yellow single-bioglue-amended clay specimen under the same content. In other words, the scouring resistance of the composite-glue-amended clay specimen was significantly better than that of the single-bioglue-amended clay specimen.

#### 3.2.2. Effect of Biogel-Modified Clays on Scour Damage Patterns

**Figure 10:**
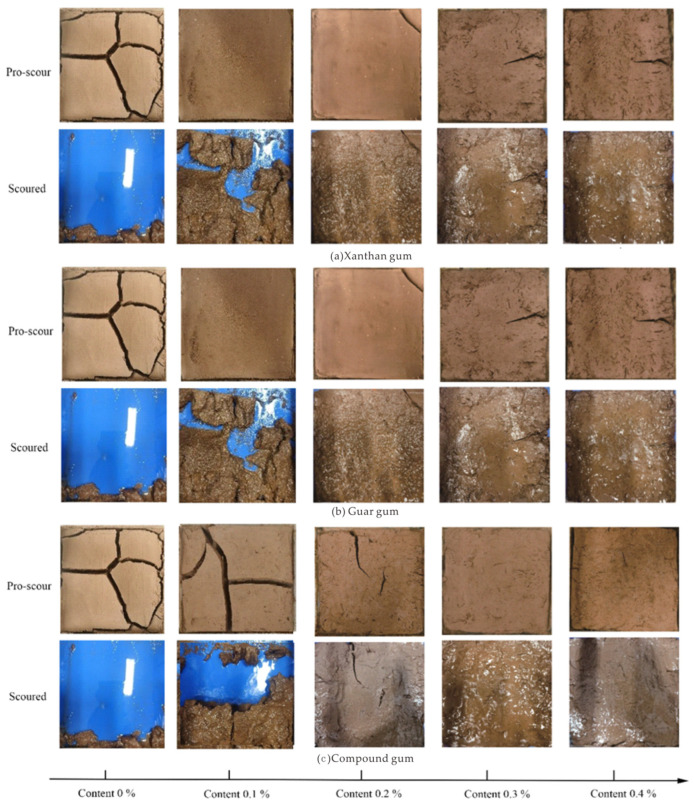
Scour damage pattern of improved clay samples with different xanthan gum contents: (**a**) xanthan gum; (**b**) guar gum; (**c**) compound gum.

**Figure 11:**
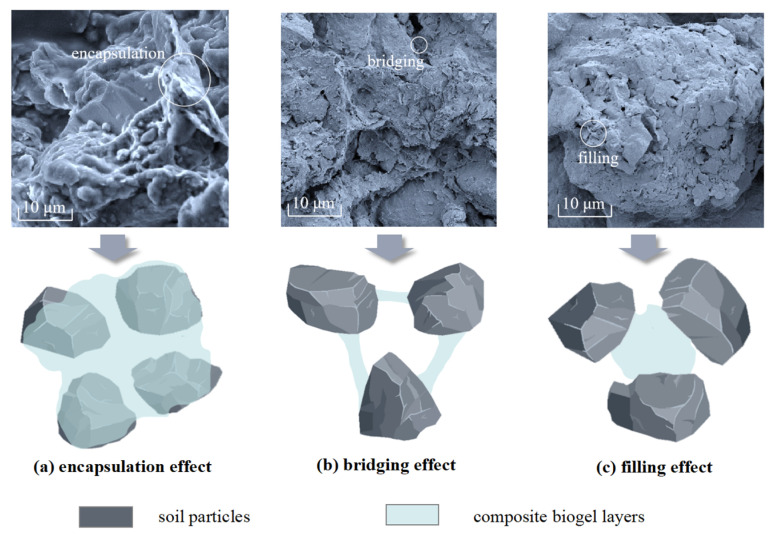
SEM image of the improved clay sample.

Figure 10a shows the modified clay before and after the change in the scouring damage pattern, in which the scouring performance of the plain soil specimens was poor, and the erosion damage was very serious. The xanthan gum improved clay scouring, and most of the block still remained in a plastic state, that is, the top surface of the specimen remained in a more complete form; only ladder-like block fissures appeared due to rainfall scouring and the erosion of the residual part. When the xanthan gum content was 0.2%, the specimen at the end of the scouring test did not appear to have clear erosion damage. Evaporation cracking appeared as larger fissures and the specimen was divided into different blocks. Under the impact of rainfall, the blocks did not appear to have clear erosion damage; only the top of the soil sample suffered from erosion and fall-off with clearer scale-like damage pattern. When the xanthan gum content was increased to 0.4%, the modified clay specimen retained very high integrity without clear erosion damage, and the destruction of the vegetal soil specimen was nearly complete under the same conditions. As shown in Figure 11a and described here, with the increase in xanthan gum content, the specimen suffered less from rain impact, the degree of surface erosion was reduced, the amount of scouring was also reduced, the scour damage resistance was further improved, the fissure edge damage was significantly reduced, and the fissures on both sides of the soil entered a fluid plastic state, causing a wide fissure reverse into small fissures.

When the specimen scouring was completed, a large amount of erosion damage occurred in the middle of the specimen, which was subjected to erosion damage in different blocks, and the surface of each block did not appear to add new cracks (Figure 10b). Combined with Figure 11, it can be seen that the scouring damage characteristics of the guar-gum-amended clay specimen are similar to those of the plain soil specimen, and are quite different from those of the xanthan-gum-amended clay, which is mainly reflected in the state of the surface of the specimen after it is damaged by erosion. Guar gum improved clay specimen particles in the rain-impacted soil with rainwater detachment and guar gum mucous membrane retained the overall integrity of the specimen. For the local soil particles, the adhesive effect was slightly lower than that of the xanthan gum mucous membrane, which made the guar-gum-improved clay specimen appear to have fewer fissures; when subjected to rain impact, soil particles were more likely to be separated from each other, and the specimen surface appeared in part of the pits. The guar gum mucous membrane was more extensively distributed in the soil than the xanthan gum mucous membrane, and the overall structural integrity of the specimen had a high degree of retention. When subjected to rainfall, the resistance of the specimen soil particles between the guar gum mucous membrane to the rainfall impact were small and appear to be subject to rainfall-impact-induced specimen damage to a greater extent. With an increase in guar gum content, the specimen suffered less from rain impact, and the degree of surface erosion was reduced. When the guar content was 0.2%, the sample surface also had a large number of pits, but the volume of pits was significantly reduced, and the number of pits slightly increased. When the guar content increased to 0.4%, the specimen did not appear to have erosion damage in the subsequent scouring test, and no visible pits were observed on the specimen surface. The guar-gum-improved clay samples and xanthan-gum-improved clay samples had similar characteristics of resistance to scouring damage and did not show clear erosion damage, and the two gums added to the clay after the formation of the bioadhesive film can result in a high degree of adhesion of soil particles in the specimen. Therefore, the specimen had higher rainfall resistance to withstand the impact of the water droplets, reducing the soil particles due to the erosion in the case of dislodgement.

The damage patterns of the specimens before and after washing with different composite biogel contents are shown in Figure 10c, which shows that the antiwashout characteristics of the composite-gel-improved clay specimen were more affected by the specimen fissure; the upper part of the specimen fissure was seriously damaged by rainfall, and the lower part of the fissure only had more pits on the surface of the specimen and did not have clear erosion damage. The effects of the single-biogel-modified clay specimen content on the fissure influence were small, and only manifested in the fissure edge part of the erosion of different degrees. Composite-gum-improved clay specimen scouring damage characteristics were similar to those of the vegetal soil specimen and guar-gum-improved clay specimen; erosion damage was observed with soil particles detached with rainwater, and the specimen surface did not appear similar to the xanthan-gum-improved clay specimens, which produced fine fissures, had damage in part of the pit, and detached from the specimen soil particles sliding down the water flow to pile up in the lower part of the specimen.

With an increase in the composite glue content, the specimen suffered less from rain impact, and the degree of erosion of the surface decreased. When the composite gum content was 0.2%, the specimen surface appeared to have a small number of pits compared to that of the composite gum content of 0.1%; the specimen erosion damage was significantly reduced, the volume and number of pits were significantly reduced, the pit state was similar to scales, the specimen fissures did not appear to have clear erosion damage, and specimen integrity was better. When the composite glue contents were 0.3% and 0.4%, there was no evident erosion damage after the scouring test, and only some erosion occurred on part of the surface of the specimen. Compared to the single biogel, the modified clay samples exhibited better erosion resistance.

### 3.3. Analysis of the SEM Test Results

Scanning electron microscopy (SEM) tests were conducted on the clay samples modified with xanthan gum, guar gum, and composite gum, and the SEM images are shown in Figure 11. The biopolymer-modified clay enhanced the anticracking and antierosion abilities of the clay samples through a series of effects.

(a) Encapsulation effect: The elastic film formed by the biopolymer in contact with water enveloped the soil particles, and the soil particles adhered to the elastic film and formed a relatively stable aggregate structure. This effectively maintained the state of the soil particles and resisted the forces exerted on them during cracking erosion.
(b) Bridging effect: The elastic film produced by the biopolymer allowed noncontacting soil particles to be interconnected, forming a relatively stable unit within a certain range. This imparted stronger cohesion to the soil particles in the sample and resisted the displacement changes caused by external forces.
(c) Pore filling: After adding the biopolymer to the soil, it formed a matrix with fine particles, reinforcing and filling the intergranular pores. This resulted in the formation of closed pores between some soil particles, preventing the ingress and egress of moisture and preventing the water content within the closed pores from evaporating. When subjected to erosive damage, external water did not penetrate and cause hydrolysis of soil particles.

## 4. Discussion

**Figure 12:**
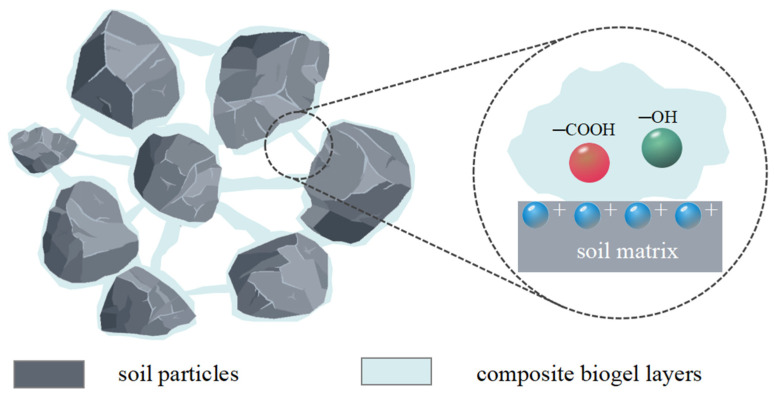
Schematic diagram of clay improved by biological glue.

A schematic diagram of the clay samples modified with biopolymers is shown in Figure 12. In the untreated soil sample, there was loose contact between the soil particles. However, after biopolymer addition, cation sorption occurred on the surface of the soil particles. The hydroxyl (–OH) and carboxyl (–COOH) groups on the biopolymer molecular chains can form ionic bonds, resulting in the bonding of the biopolymer to the surfaces of the soil particles. The elastic films of the biopolymer adsorbed onto different soil particles come into contact and interconnect with each other, promoting closer contact between the soil particles and enhancing the cohesive bonding between them.

When the biopolymer content was low, the elastic film formed failed to effectively bridge the soil particles, resulting in only a small fraction of agglomerates in the sample and the absence of effective bridging. As the biopolymer content increased, the elastic film diffused among the soil particles, forming a stable three-dimensional network structure. This structure can effectively encapsulate and bridge soil particles, fill the void between them, and promote closer contact between the particles. The biopolymer film significantly enhanced the force-resistant deformation between the soil particles. When external forces act on soil particles, the elastic film redistributes a large portion of the tensile force between the particles. Consequently, when displacement occurs, the elastic film is the first to break. As a result, the tensile strength of the sample was significantly improved, and its resistance to cracking and erosion was enhanced.

**Figure 13:**
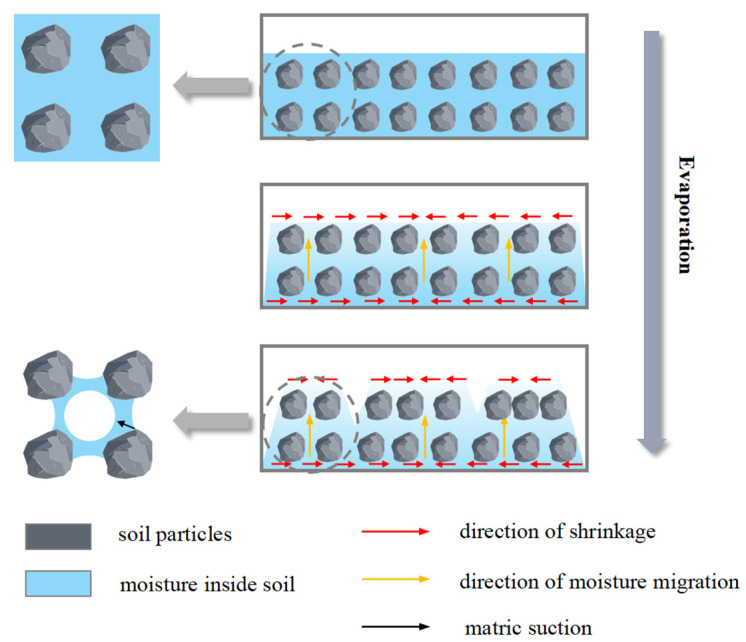
Schematic diagram of specimen cracking.

Owing to the presence of a bound hydration film on the surface of the soil particles, direct contact between the particles is hindered, which provides space for contraction during soil desiccation. Therefore, during drying and water loss, free water on the soil surface evaporates, and water in the lower layers of the soil migrates upward under capillary action to compensate for evaporation at the surface, resulting in a decrease in the pore radius and the development of a tensile stress field, as shown in Figure 13. When the tensile stress at a certain point within the soil exceeds its tensile strength, tensile rupture occurs, forming V-shaped cracks. However, after incorporating the biopolymer into the soil, it formed a matrix with fine particles, strengthening and solidifying the soil while filling interparticle voids. This hindered the upward movement of water from the lower layers to compensate for surface evaporation, reduced and prolonged the water migration path, and slowed water evaporation. This reduction in surface tension, along with enhanced interparticle bonding, decreased the porosity and strengthened the integrity of the soil, thereby improving its tensile strength. With an increase in the biopolymer content, the connection between the biopolymer and soil particles became tighter, resulting in a more stable soil structure and enhanced resistance to cracking.

**Figure 14:**
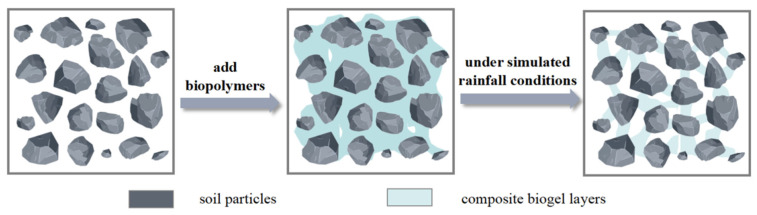
Schematic diagram of inter-particle erosion behavior.

Figure 14 illustrates the biopolymer-modified clay samples under erosion. The biopolymer formed a gel layer after being added to the clay, which enveloped the soil particles and enhanced cohesion between adjacent particles. Moreover, biopolymers can bind to a large number of soil particles, ensuring their relative stability and improving the integrity of the sample. As a result, the sample exhibited better resistance to water droplet impacts during erosion by rainfall and runoff, which reduced the occurrence of soil particle detachment. Additionally, the biopolymer matrix impeded the downward seepage of water, effectively enhancing the resistance of the sample to erosion.

Among the modified clay samples with the same biopolymer content, the guar-gum-modified clay exhibited a higher degree of encapsulation and cohesion of soil particles than those modified with xanthan gum. This effectively combined a greater number of soil particles into a cohesive mass, resulting in enhanced stability. The composite gel, owing to its unique synergistic enhancement effect, improved the bonding strength between the soil particles, increased soil cohesion, and enhanced mechanical strength. Microscopically, the composite gel tightly enveloped and connected the soil particles, which significantly improved the overall integrity of the sample and its resistance to disintegration.

**Figure 15:**
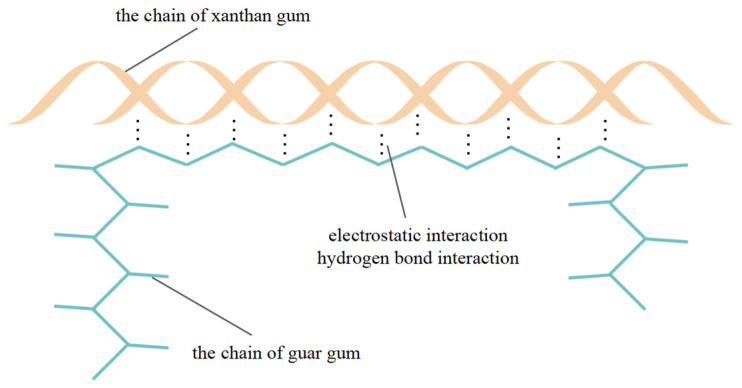
Schematic diagram of synergistic effect between xanthan gum and guar gum.

The composite gum was a mixture of xanthan gum and guar gum in a ratio of 1:1. The synergistic effect of xanthan gum and guar gum has been widely used in the food industry; however, the mechanism underlying this synergistic effect has been controversial in recent years. It is generally believed that a synergistic effect is achieved through synergistic forces such as electrostatic action and hydrogen bonding [26,27,28], as shown in Figure 15. Electrostatic interactions are the main forces that form polymer complexes. Electrostatic interactions between natural polymers can be affected by changing the charge density or introducing metal ions with a shielding effect. Hydrogen bonding has a significant effect on the aggregation state, structure, and physical properties of substances; the most significant effect is that it can improve the stability of the system. The molecular chain of xanthan gum forms a spiral structure in solution, further improving the stability of the gel, and the functional groups on the molecular side chain exhibit a good adhesion effect. Galactose in guar gum and xanthan gum forms a more stable three-dimensional network structure after spiral combination, so that the soil particles in the sample can be more effectively bonded together, and the resistance to erosion is enhanced.

In samples with the same concentration, the mass of each biopolymer in the composite gel was 50% of that in single biopolymer samples with the same concentration. Owing to the synergistic enhancement effect of the two biopolymers, the composite gel was unable to adequately encapsulate and bond the soil particles at lower concentrations, resulting in poorer antidisintegration characteristics when the concentration was below 1%. However, once the concentration reached 1%, the composite gel fully and effectively encapsulated the soil particles, allowing the sample to bond together as a solid mass and significantly improving its antidisintegration properties.

The addition of biogum effectively promotes the ecological restoration effect. Previous studies have shown that the soil treated with xanthan gum and guar gum had the superior vegetation germination rate [29,30]. The superior performance can be attributed to the improved water retention capacity due to their hydrophilicity and adhesion. Thus, the addition of biogum offers an advantageous plant growth environment, effectively preventing clay from cracking and reducing erosion, which contributes to slope stability.

## 5. Conclusions

In this study, we comprehensively investigate the water retention, cracking resistance, and scouring resistance of biogel-enhanced clays by examining their hydrological properties and performing evaporation cracking and scouring tests. The key findings are as follows:

1. The addition of biogel to clay improved the water retention properties of clay; clay with a lower biogel content had a lower average evaporation rate in the uniform evaporation stage, and the final evaporation of composite-biogel-modified clay was lower than that of single-biogel-modified clay. With an increase in biogel content, the average evaporation rate in the uniform evaporation stage decreased, and the onset of the deceleration and stabilization evaporation stages was delayed to some extent. This effect was most significant when the biogel content reached 0.4%.
2. The incorporation of biogels improved the anticracking performance of the samples. With an increase in the biogel content, the number of cracks and degree of crack development in the specimen were significantly reduced. With the evaporation of water from the specimen, the specimen contracted significantly. Different content of xanthan gum improved clay sample cracking to a different degree; guar gum improved clay sample anticracking properties more than xanthan gum, and at higher gum content, larger cracks did not occur; composite-gum-improved clay samples with a content of more than 0.2% did not produce clear cracks.
3. The addition of biogum significantly improved the scour resistance of the clay specimens. The abrasion resistance of the clay specimens modified with xanthan gum was better than that of the clay specimens modified with guar gum. The abrasion resistance of the composite-gum-amended clay specimens was significantly higher than that of a single biogum. With the incorporation of biogum, the degree of abrasion damage of the samples was reduced. When the biogum content reached 0.2%, the specimens did not show any clear abrasion damage, demonstrating the beneficial effects of incorporating biogel into clay, particularly in terms of water retention, crack resistance, and scour resistance. This study highlights the potential applications of improved biogel clays in various fields.

## Figures and Tables

**Table 1:** Results of chemical composition analysis of vegetal soil.

Compound Name	SiO_2_	Al_2_O_3_	CaO	Fe_2_O_3_	MnO	MgO	Na_2_O	K_2_O	Other
Mass percentage (%)	61.12	14.60	10.74	2.15	2.01	1.96	1.74	1.64	4.04

**Table 2:** Basic parameters of xanthan gum.

Item	CAS No.	Melting Point (°C)	Boiling Point (°C)	Granularity mm	pH	Ash Content (%)	Drying Loss (%)	Viscosity (MPa·s)
Information	11138-66-2	64.4	180.0	<0.18	7.0	≤13.0	≤15.0	>600

**Table 3:** Basic parameters of guar gum.

Item	CAS No.	Odor	Stability	Melting Point (°C)	Specific Spin	Ash Content (%)	Drying Loss (%)	Viscosity (MPa·s)
Information	9000-30-0	Slightly odorous	Stable	>220	D25 + 53°	≤1.5	≤12.0	>300

## Data Availability

The data presented in this study are available on request from the corresponding author.
